# A prognostic gene model of immune cell infiltration in diffuse large B-cell lymphoma

**DOI:** 10.7717/peerj.9658

**Published:** 2020-08-05

**Authors:** Hao Zhou, Chang Zheng, De-Sheng Huang

**Affiliations:** 1 Department of Epidemiology, School of Public Health, China Medical University, Shenyang, Liaoning, China; 2 Department of Impression Evidence Examination Technology, Criminal Investigation Police University of China, Shenyang, Liaoning, China; 3 Key Laboratory of Cancer Etiology and Prevention (China Medical University), Liaoning Provincial Department of Education, Shenyang, Liaoning, China

**Keywords:** Diffuse large B-cell lymphoma, Immune cells, Immunoscore, Tumor microenvironment, Natural killer cell, CIBERSORT algorithm, Lasso regression

## Abstract

**Background:**

Immune cells in the tumor microenvironment are an important prognostic indicator in diffuse large B-cell lymphoma (DLBCL). However, information on the heterogeneity and risk stratification of these cells is limited. We sought to develop a novel immune model to evaluate the prognostic intra-tumoral immune landscape of patients with DLBCL.

**Methods:**

The ESTIMATE and CIBERSORT algorithms were used to estimate the numbers of 22 infiltrating immune cells based on the gene expression profiles of 229 patients with DLBCL who were recruited from a public database. The least absolute shrinkage and selection operator (Lasso) penalized regression analyses and nomogram model were used to construct and evaluate the prognostic immunoscore (PIS) model for overall survival prediction. An immune gene prognostic score (IGPS) was generated by Gene Set Enrichment Analysis (GSEA) and Cox regression analysis was and validated in an independent NCBI GEO dataset (GSE10846).

**Results:**

A higher proportion of activated natural killer cells was associated with a poor outcome. A total of five immune cells were selected in the Lasso model and DLBCL patients with high PIS showed a poor prognosis (hazard ratio (HR) 2.16; 95% CI [1.33–3.50]; *P* = 0.002). Differences in immunoscores and their related outcomes were attributed to eight specific immune genes involved in the cytokine–cytokine receptor interaction and chemokine signaling pathways. The IGPS based on a weighted formula of eight genes is an independent prognostic factor (HR: 2.14, 95% CI [1.40–3.28]), with high specificity and sensitivity in the validation dataset.

**Conclusions:**

Our findings showed that a PIS model based on immune cells is associated with the prognosis of DLBCL. We developed a novel immune-related gene-signature model associated with the PIS model and enhanced the prognostic functionality for the prediction of overall survival in patients with DLBCL.

## Introduction

Diffuse large B-cell lymphoma (DLBCL) is the most common aggressive non-Hodgkin lymphoma worldwide (Perry et al., 2016). DLBCL is highly heterogeneous in terms of genetic findings, clinical course, response to therapy, and prognosis (Reddy et al., 2017). Next generation sequencing (NGS) has clarified the heterogeneity and identified a variety of genetic alterations for risk stratification of patients with DLBCL (Chapuy et al., 2016; Karube et al., 2018; Arthur et al., 2018; Schmitz et al., 2018). Recently, several prognosis predictors such as the international prognostic index (IPI) and gene mutations, have demonstrated greater ability to stratify the risk of patients with DLBCL (Cabanillas & Shah, 2017). The personalized treatment approach of targeting oncogene addiction may be more precise compared with chemoimmunotherapy but is associated with increased drug resistance (Reddy & Thieblemont, 2017). Most therapeutic strategies directly targeted tumor cells; however, stromal and immune cells in the tumor microenvironment (TME) are genetically stable and less susceptible to therapeutic resistance (Klemm & Joyce, 2015).

Cancer cells are overwhelmingly genetically heterogeneous. A large body of molecular evidence suggests that TME interactions represent potential points for therapeutic strategies (Quail & Joyce, 2013). TME-reprogrammed host stromal and immune cells including myeloid cells, B cells, T cells, and malignantly transformed cells (Scott & Gascoyne, 2014). Using the numbers and types of immune cells infiltrating the TME combined with tumor- and TME-associated characteristics will enable a more precise stratification of DLBCL cases to determine more accurate prognoses (Miao et al., 2019). CIBERSORT is a suitable machine-learning algorithm that can reconstruct the immune cell subsets infiltrating the TME based on gene expression profiles of the cell mixtures (Gentles et al., 2015). CIBERSORT can identify bias produced by traditional methods, such as immunohistochemistry and flow cytometry (Cherian, Hedley & Keeney, 2019; Craig & Foon, 2008), and provide the ratio of immune cells to determine the infiltrating cellular components of the TME.

We used the Estimation of STromal and Immune cells in Malignant Tumors using Expression data (ESTIMATE) (Yoshihara et al., 2013) and CIBERSORT algorithms to identify the quantities and ratios of 22 unique, prognosis-associated human immune cells in the TME of 229 DLBCL tissues. Least absolute shrinkage and selection operator (Lasso) Cox regression analyses and nomograms were used to establish a prognostic immunoscore model. This model was used to stratify patients with DLBCL into high-risk and low-risk groups for detecting biological functions and key genes to predict survival. We also developed and validated a novel immune-related gene-signature for prognostication of DLBCL patients.

## Materials & Methods

### Data preparation for CIBERSORT

All genome data in this study originated from public databases and the workflow analysis is presented in Fig. S1. Statistical analyses were conducted using R v3.5.1 and Bioconductor v3.8 (https://www.bioconductor.org/). Illumina HiSeq RNASeq RNA expression data of lymph nodes for patients diagnosed with DLBCL with clinical information (*N* = 229) were downloaded from the National Cancer Institute (NCI) Center for Cancer Research (CCR) of the Cancer Genome Atlas (TCGA, https://tcga-data.nci.nih.gov/tcga/) program. RNA-sequencing data in HTSeq-counts type were downloaded and converted into gene symbols by the Genome Reference Consortium Human Build 38 patch release 12 (GRCh38.p12) of Ensembl. We removed genes with counts of < 10 to prepare the expression data profiles for CIBERSORT analysis then counts were normalized using voom (R package limma).

The ESTIMATE algorithm was performed to evaluate the tumor purity of each patient by presenting the infiltration of immune and stromal cells using the expression data. The algorithm was based on a single sample Gene Set Enrichment Analysis (ssGSEA). The estimated score, generated by the ssGSEA algorithm and the R script followed as estimateScore (input.ds = “commonGenes.gct”, output.ds = “estimateScore.gct”, platform = “illumina”) were the sum of the immune and stromal scores that indicated tumor purity. Patients with a percent composition of tumor purity >0.6, which distinguished tumor and non-tumor tissue (immune and stromal), were eligible for further analysis (Rhee et al., 2018).

### Profiles of tumor-infiltrating immune cells and prognoses in DLBCL

The CIBERSORT parameters (https://cibersort.stanford.edu) with the gene signature matrix (LM22) were used as a reference for comparison to quantify the proportions of tumor-infiltrating immune cells in DLBCL tissues (Newman et al., 2015). The number of permutations was set at 1,000 with random lists to the smallest subset used to define any one of the 22 types immune cells. The LM22 matrix contains 547 genes defining 22 immune cell phenotypes: naïve and memory B cells, CD8+ T cells, naïve CD4+ T cells, resting and activated CD4+ memory T cells, regulatory T cells (Tregs), follicular helper T cells (Tfh), γδ T cells, M0, M1, and M2 macrophages, plasma cells, resting and activated mast cells, resting and activated natural killer (NK) cells, resting and activated dendritic cells, monocytes, eosinophils, and neutrophils.

The distribution of these 22 subtypes of immune cells in each patient with DLBCL was determined by the CIBERSORT R script v1.03. The correlation coefficient, *P* value, and root mean squared error were also determined. A *P*-value of ≤ 0.05 rendered samples eligible for further analysis.

### Lasso model establishment of immunoscores

Lasso Cox regression analysis was used to select the significant prognostic features of 22 immune cells and estimate the likelihood of deviance. In the TCGA cohort, features of immune cells for predicting overall survival (OS) were based on relative immune cell fractions and the coefficients were used to develop the Lasso model. The K-fold cross-validation was set as 10-fold and the best tuning parameter (lambda) value as the minimum value of lambda. A value of 0 was assigned when the fraction level of the immune cell was higher than the corresponding cut-off value, otherwise, a value ≤ 0 was present. Lasso Cox regression was calculated with the glmnet package in R (Friedman, Hastie & Tibshirani, 2010). The script was glmnet (x, y, family = “cox”, alpha = 1, nlambda = 100) and cv.glmnet (x, y, family = “cox”, type=“class”, nfolds = 10). Five types of prognostic immune cells for OS were ultimately selected to establish the tumor-infiltrating prognostic immunoscores (PIS). Whole sample sets were divided into two risk groups based on the PIS calculated by Lasso regression (Fu et al., 2018): low-PIS was defined as PIS ≤ 0 and high-PIS groups as >0.

### Nomogram construction and validation

Clinicopathological (age, sex, clinical stage, and IPI) characteristics and immune score types were used to develop the nomogram using the multivariable Cox proportional hazard model based on the nomogram R software package. All of the potential risk factors in the multivariate analysis were applied to construct a nomogram model for predicting survival. The 1-, 3-, and 5-year discriminations of the nomogram were measured by Harrell’s concordance index (C-index) and by calibration plot, based on R script calibrate (model, cmethod = ‘KM’, method = “boot”, u = days, *m* = 70, *B* = 217) to calculate the sensitivity of diagnostic and predicted survival probabilities, respectively.

### Prognostic analysis

OS rates of immune cells or genes were analyzed using the univariate Kaplan–Meier method and the corresponding significance of the survival curves was evaluated using the log-rank test. A hazard ratio (HR) with a 95% confidence interval (95% CI) was calculated by multivariate Cox proportional hazard models to quantify the strength of the association between relevant parameters and overall survival. Comparison between immune cells and genes expression was stratified into high- and low-levels based on median value. All statistical tests were performed by survminer package (https://CRAN.R-project.org/package=survminer) as two-sided. *P*-values < 0.05 were considered statistically significant.

### Gene set enrichment analysis

Gene set enrichment analysis (GSEA) was performed to identify potential biological functions by categorically labeling the samples according to the stromal immune score type (Subramanian et al., 2005). We tested approximately 1,500 gene sets from molecular signatures databases (c2.cp.kegg.v7.0.symbols and c5.bp.v7.0.symbols) using GSEA v3.0 based on JAVA v8.0 script. The number of random sample permutations was set at 1,000 and the selection threshold was based on value of false discovery rate (FDR). Both biological process and KEGG pathways with FDR *q* value < 0.1 was used as a cutoff for inclusion.

### Development of gene predictive score

Key immune genes were identified. Genes for biological process and KEGG pathways were enriched by GSEA enrichment and were differentially expressed between high- and low-PIS groups. The univariate Cox regression analysis was ultimately performed to detect the association with prognosis of DLBCL. The edgeR package (http://bioconductor.org/packages/edgeR/) was used to identify differentially expressed genes between high- and low-PIS groups. A statistically significant FDR value of 0.05 and absolute value of log2 fold change ≥ 1.2 was defined as differentially expressed genes. These filtering criteria led to the identification of eight key genes. A weighted formula was applied to develop the key immune gene prognostic score (IGPS) and enhance the prognostic ability of the eight key genes. The weight was generated by combining each gene’s HR and expression level.

### Validation in the GEO database

To minimize bias, the Gene Expression Omnibus (GEO, https://www.ncbi.nlm.nih.gov/geo) (ID: GSE10846) dataset were used to confirm that the proposed PIS and IGPS model had a similar prognostic value in a different DLBCL population. The same formula was applied to the validation cohort. Clinical data of survival and gene expression were downloaded and normalized from the GEO database.

## Results

### Profiles of tumor-infiltrating immune cells and prognoses in DLBCL

**Figure 1:**
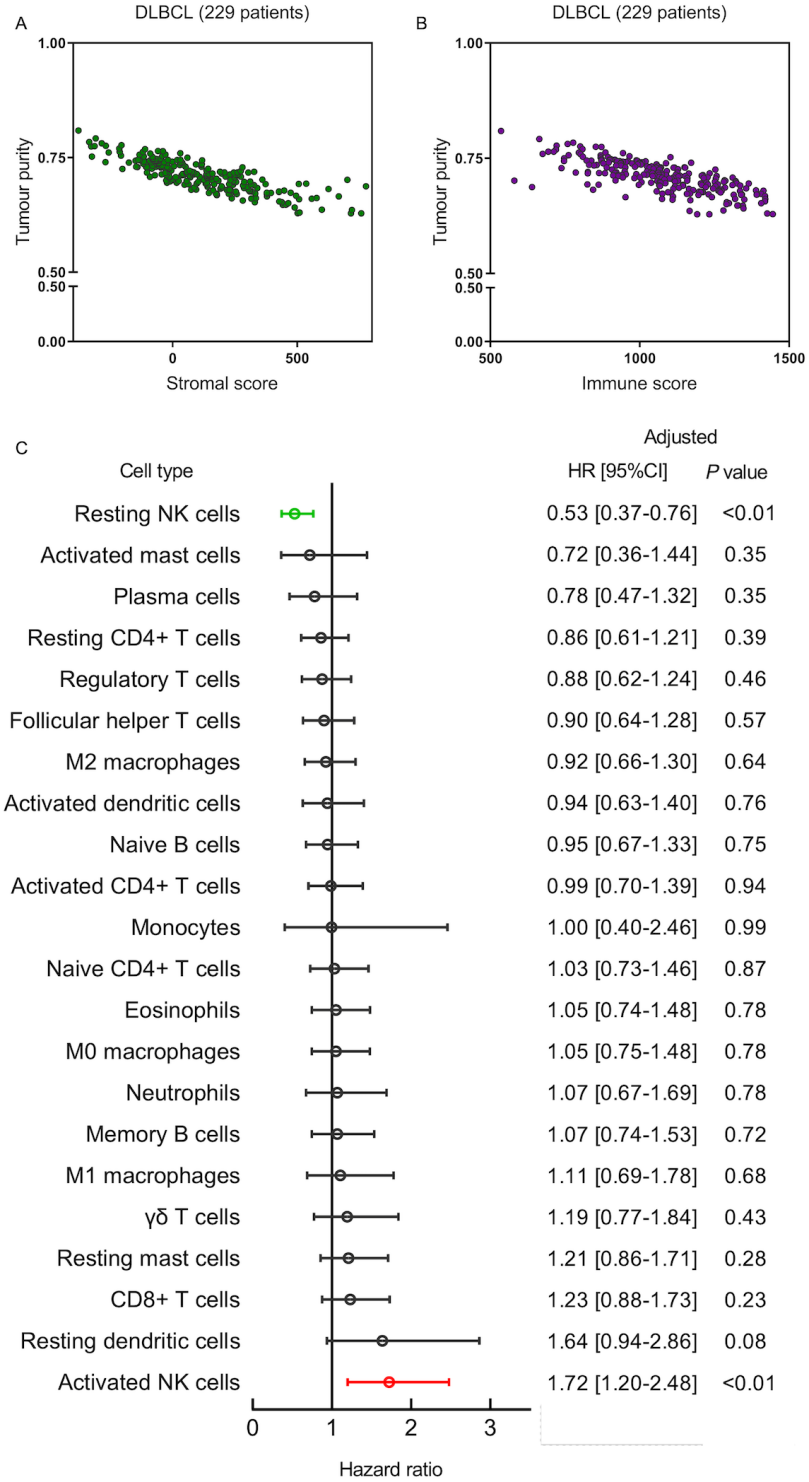
The prognostic value of tumor-infiltrating immune cells in diffuse large B-cell lymphoma (DLBCL). Scatterplots between tumor purity and stromal (A) and immune (B) scores in the 229 TCGA DLBCL patients. (C) Forest plot showing the hazard ratios of the 22 human immune cells to the overall survival benefits in DLBCL. HR, hazard ratio; CI, confidence interval; adjusted by age, gender, IPI score and clinical stage.

Two hundred and twenty-nine patients with DLBCL and OS data were studied. Patient characteristics are detailed in Table 1. The median age at diagnosis was 60 years (range 16–90 years) and 93 (40.6%) of the patients were female. ESTIMATE was applied before the detection of the relative abundance of 22 immune cells to examine the overall abundance of stromal and immune cells to predict tumor purity (Figs. 1A and 1B). The tumor purity of all 229 DLBCL patients was greater than 60%, offering a convincing result for subsequent analysis (Table S1). The abundance ratios of 22 immune cell types present in the TME were evaluated in each of the patients with DLBCL using the CIBERSORT algorithm. Memory (22.6%) and naïve (16.1%) B cells were the most abundant immune infiltrates in DLBCL (Fig. S1), followed by tumor-related macrophages (M0 11.7%, M2 8.2%, and M1 7.4%), CD4+ (10.3%) and CD8+ (8.2%) T cells, resting (2.3%) and activated (0.6%) NK cells, and Tregs (1.4%). The sum abundant of these tumor-infiltrating immune cells were commonly observed in DLBCL (Fig. S2).

**Table 1:** Characteristics of 229 DLBCL patients in TCGA datasets.

Variable		Number of patients	%
Age at diagnosis (years)	<60	112	48.9
	≥60	117	51.1
Gender	Female	93	40.6
	Male	136	59.4
Stage (Ann Arbor)	I+II	109	47.6
	III+IV	120	52.4
IPI score distribution	Low (0, 1 and 2)	126	55.0
	Intermediate-high and high (≥3)	103	45.0

**Notes.**

IPI, international prognostic index.

Based on the TCGA dataset, a total of 22 immune cell phenotypes infiltrating the TME were used as potential prognostic markers for DLBCL (Fig. 1C). The survminer R package was used to determine the best cut-off values for each immune cell fraction to predict clinical outcomes of DLBCL. Kaplan–Meier curve analysis and log-rank test were used to determine that the cases with a higher proportion of activated NK cells in the TME showed significantly shorter OS (*P* < 0.001), whereas resting NK cells were more representative of the TME of patients with better outcomes (*P* = 0.001). The association of OS and each TME-associated immune cell type was also analyzed by the multivariate Cox hazard regression, adjusted by age, gender, IPI score, and clinical stage (Altman et al., 1994). The activated and resting NK cells were independent risk factors for DLBCL prognosis with a significant adjusted HR of 1.72 (95% CI [1.20–2.48]; *P* = 0.001) and 0.53 (95% CI [0.37–0.76]; *P* = 0.005) (Fig. 1C). Poor prognosis was associated with increased TME infiltration of activated NK cells and reduced TME numbers of resting NK cells in DLBCL samples.

### Establishment of a prognostic immunoscore model for DLBCL

**Figure 2:**
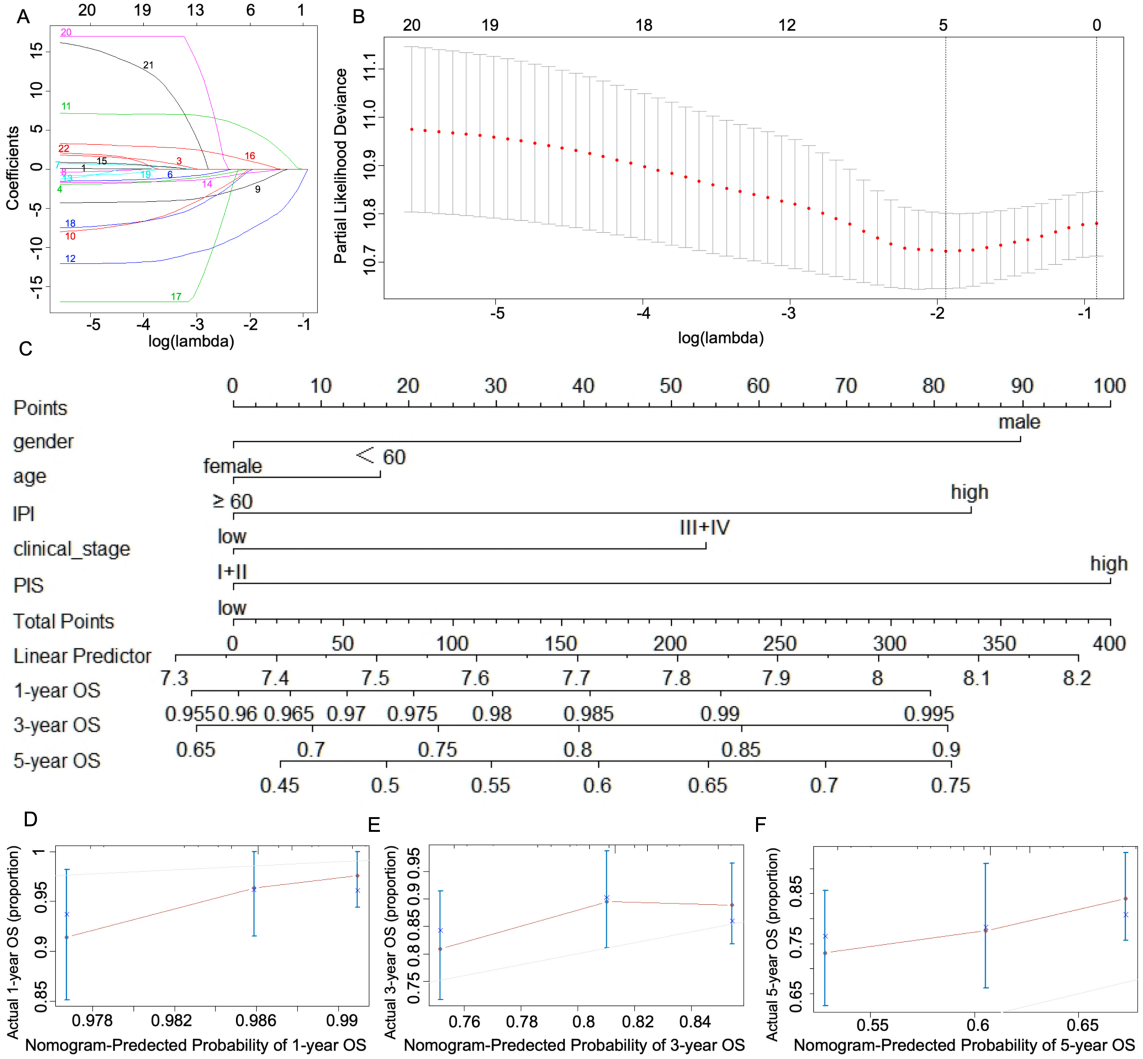
Construction and validation of the DLBCL immune score model. (A) Lasso coefficient profiles for the fractions of 22 immune cells. Immune cell type: 1, naïve B cells; 2, memory B cells; 3, plasma cells; 4, CD8+ T cells; 5, naïve CD4+ T cells; 6, resting CD4+ memory T cells; 7, activated CD4+ memory T cells; 8, follicular helper T cells; 9, regulatory T cells; 10, *γδ* T cells; 11, resting NK cells; 12, activated NK cells; 13, monocytes; 14, M0 macrophages; 15, M1 macrophages; 16, M2 macrophages; 17, resting dendritic cells; 18, activated dendritic cells; 19, resting mast cells; 20, activated mast cells; 21, eosinophils; 22, neutrophils. (B) The tuning parameter (Lambda) selection in the Lasso model. The red dots represent the partial likelihood deviance values, with the gray lines representing the error bars. The dotted vertical lines are drawn at the optimal values by minimum criteria and 1-s.e. criteria. In A and B, the numbers above the graph represent the number of cell types involved in the Lasso model. (C) Nomogram for predicting 1-, 3- and 5-year overall survival of DLBCL patients based on gender, age, clinical stage, IPI, and immune score types. Calibration curves of predicted and observed outcomes of 1-(D), 3-(E) and 5-(F) year nomograms.

Lasso penalized Cox regression was used to build a microenvironment immune score model in DLBCL according to the minimum value of lambda as 0.14 and partial likelihood deviance of 10.72042. As we focused on the comprehensive tumor microenvironment of DLBCL, the Lasso regression model were conducted by a total of 22 immune cells instead of immune cells with significant prognosis. According to the iterative shrinkage threshold of regularization (Fig. 2A) and the trajectory of each goodness of fit (Fig. 2B), only five types of immune cells (activated and resting NK cells, Tregs, and M0 and M2 macrophages) were included to build the microenvironment immune score. The Lasso formula is as follows: PIS = 7.51*activated NK cells - 4.81*resting NK cells + 1.87*Tregs + 0.96*M2 Macrophages - 0.41*M0 Macrophages. By using this formula, PIS were determined for a total of 229 patients with DLBCL, who were then stratified into risk groups. High-PIS was defined as PIS >0 and low-PIS defined as PIS <0. The patients in the high-PIS group showed a significantly poor prognosis, HR = 2.16 (95% CI, [1.33 −3.50]; *P* = 0.002) compared with those in the low-PIS group.

To evaluate the prognostic value of the newly defined microenvironment immunotype, a nomogram model was built to predict the probability of 1-, 3-, and 5-year OS in patients with DLBCL. The risk variables were integrated by the immunoscore and clinicopathology (age at diagnoses, sex, IPI, and clinical stage). Table 2 shows the results of the Cox multivariate analysis. Two variables were independently associated with significant OS, high immune score (HR: 1.95, 95% CI [1.20–3.17]) and IPI score (HR: 2.24, 95% CI [1.21–4.15]), which increased the risk of reduced survival approximately twofold, compared with a low score in PIS and IPI repectively. The nomogram demonstrated that the PIS groups contributed the most to the prognosis, moreso than the clinical stage or IPI (Fig. 2C). To assess the predictive ability of the nomogram, a calibration plot and C-index were performed for the probability of 1-, 3-, and 5-year OS and the C-index value was 0.6 (Fig. 2D). Our nomogram model provided a better predictive accuracy of DLBCL.

**Table 2:** Cox multivariate analysis of clinicopathological parameter of 229 DLBCL patients in nomogram model.

Variable		N	Multivariate Cox regression	
			Hazard ratio (95% CI)	*P*
Gender	Female	93		
	Male	136	1.12 (0.73–1.71)	0.618
Age	<60	112		
	≥60	117	1.48 (0.95–2.30)	0.086
Clinical stage	I+II	120		
	III+IV	109	1.08 (0.66–1.76)	0.752
IPI score	Low (0, 1 and 2)	126		
	High (≥3)	103	2.24 (1.21–4.15)	0.011^*^
Immune score	Low	77		
	High	152	1.95 (1.20–3.17)	0.007^*^

**Notes.**

Not all cases initially included in the study are included as some patients failed to provide the relevant clinical parameters.

* *P* < 0.05.

IPI international prognostic index N number

### Identification of PIS-associated biological implications and modulators

**Figure 3:**
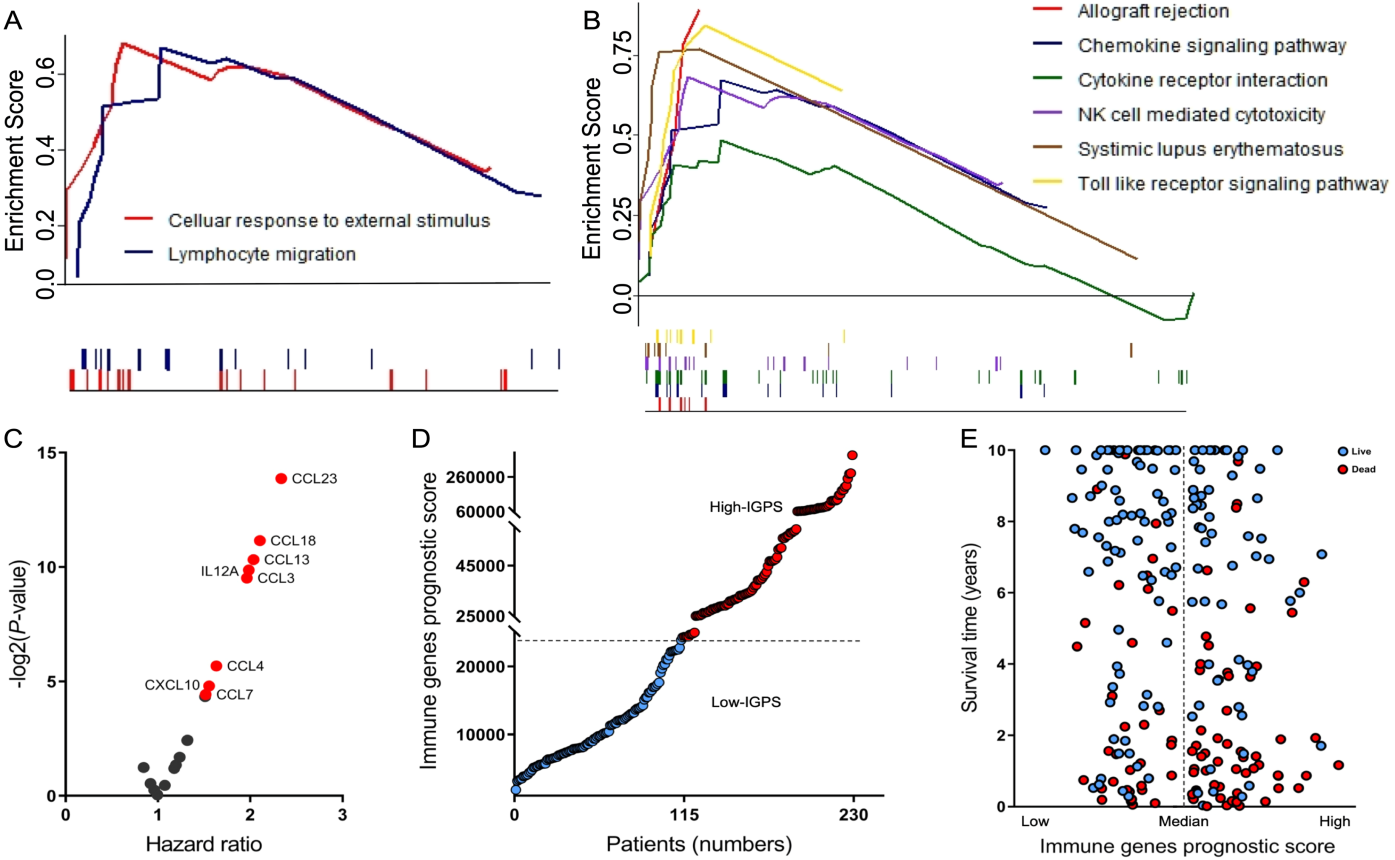
Biological functions and key genes of PIS. GSEA results based on differentially genes showing the biological process (A) and KEGG pathway (B) associated with PIS type. The top portion of each plot shows the running enrichment score, in which the peak value represents the ES. The bottom portion shows where each gene appears in the ranked list of genes. Each gene is marked as a vertical line with the corresponding color. In these genes’ enrichment sets, the leading edge subset appears subsequent to the peak positive enrichment score. (C) Volcano plot of key genes for overall survival prediction in DLBCL. The red dots represent highly expressed genes significantly correlated with poor outcomes. (D) A dot plot shows the distribution of IGPS in 229 DLBCL patient sets. (E) A dot plot shows the distribution of IGPS with clinical outcomes in 229 DLBCL patients (blue, patients alive; red, patients dead). IGPS, immune genes prognosis score.

GSEA analyses indicated that the high-PIS subgroup was highly enriched in two biological processes: cellular response to external stimulus and lymphocyte migration (Fig. 3A). Furthermore, KEGG pathway enrichment analysis established that the types of genes expressed in patients of the high-PIS subgroup were involved in six pathways: chemokine signaling pathways, toll-like receptor (TLR) signaling pathways, cytokine–cytokine receptor interactions, allograft rejection, NK cell mediated cytotoxicity, and systimic lupus erythematosus (Fig. 3B). The lines in the upper portion of each plot showed the enrichment score, reflecting the degree to which the gene set is over-represented at the top of the ranked list of genes. Each gene is marked as a vertical dashed lines underneath the plots, with the core gene members that contribute most to the ES are called the leading edge subset genes. Eighteen key genes were cross-analyzed by enrichment in the biological processes and KEGG pathways (Table S2).

We then detected differentially expressed genes between the high- and low-PIS subgroups. A total of 18 key immune genes were differentially expressed genes (log fold change ≥ 1.2 and *P*-value < 0.05) with 11 upregulated and 7 downregulated in high-PIS groups compared with low-PIS. In addition, univariate log-rank and Cox regression analyses were performed to determine the prognosis value of key immune genes. We identified eight genes [encoding chemokine (C-C motif) ligand (CCL)18, CCL4, CCL3, CCL23, CCL7, CCL13, chemokine (C-X-C motif) ligand (CXCL)10, and interleukin (IL)-12A] that were significantly elevated in the high-PIS subgroup. Their high expression levels predicted a shorter OS than the low-PIS group (Fig. 3C and Table S3).

We combined these eight genes into one signature of IGPS to further strengthen the predictive ability of the key immune genes for each patient using the weighted formula: IGPS score for each patient = (1.96*expression of CCL3)+(1.63*expression of CCL4)+(1.52*expression of CCL7)+(2.04*expression of CCL13)+(2.11*expression of CCL18)+(2.34*expression of CCL23)+(1.56*expression of CXCL10)+(1.98*expression of IL12A). The IGPS distribution is presented in Fig. 3D. We divided the patients into high and low IGPS groups based on the median value of IGPS. Patients with higher IGPS showed greater mortality on the dot plot of Fig. 3E. We validated the prognostic utility of the IGPS score in a univariate Cox regression analysis (HR: 2.14, 95% CI [1.40–3.28], Table S3). As expected, IGPS was found to be an independent and superior predictor of survival in DLBCL patients.

### Validation in GSE10846 dataset

**Figure 4:**
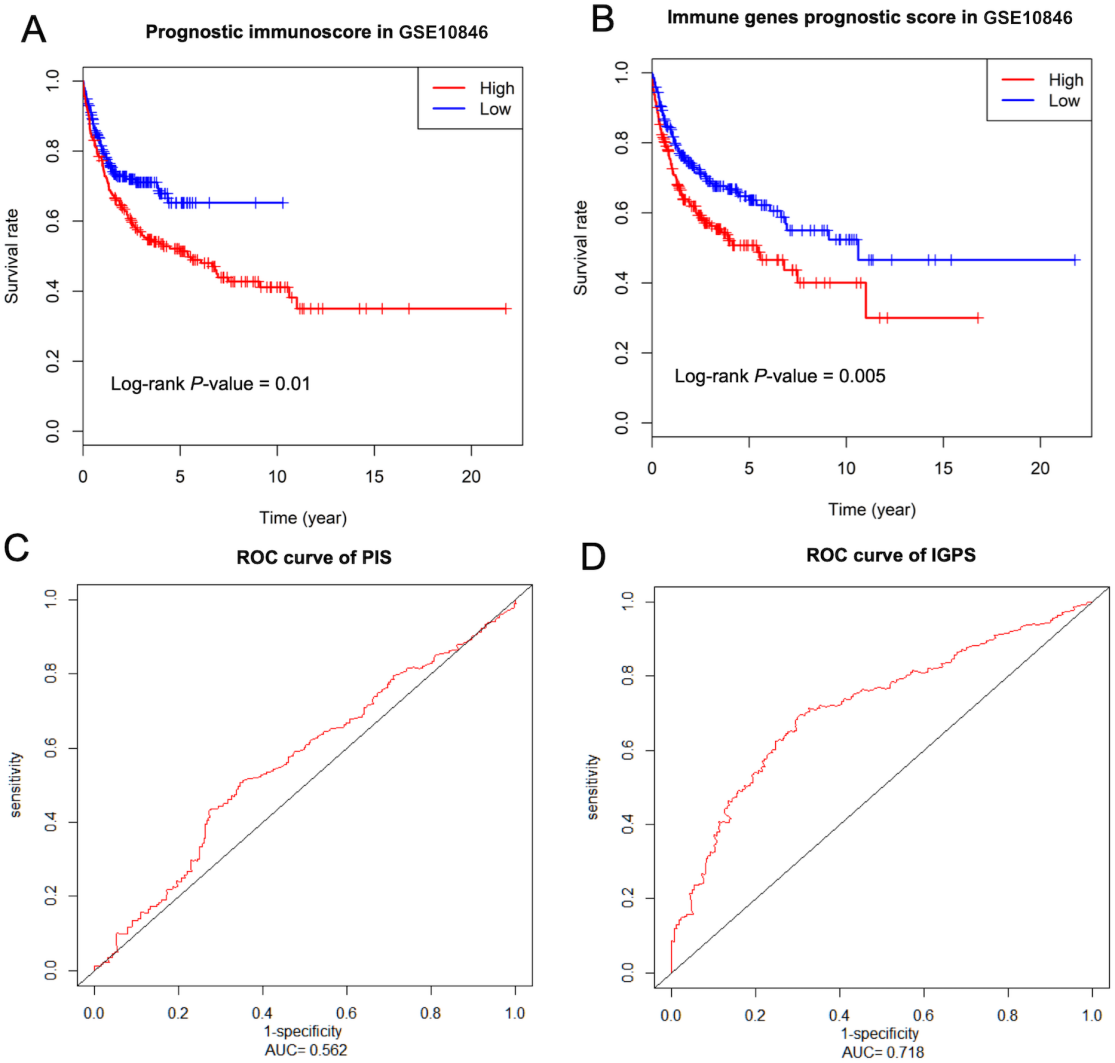
Validation in the GSE10846 dataset. Kaplan–Meier curves show survival differences in patients belonging to high and low PIS (A) or IGPS (B) groups. The corresponding ROC curve for PIS (C) or IGPS (D) groups, 10 years as a cutoff value in the GSE10846 dataset. PIS, prognostic immunoscore; IGPS, immune genes prognosis score.

The dataset GSE10846 was used to validate our earlier analysis. The GSE10846 dataset contained the clinical and gene expression data of 414 DLBCL patients, which was determined by Affymetrix Human Genome U133 Plus 2.0 (HG-U133 Plus_2.0). First, we identified 22 types of immune cells. Then, the Kaplan–Meier curves suggested that the patients in the high PIS or IGPS group had a significantly poor prognosis based on the log-rank test (Figs. 4A and 4B). The corresponding ROC curves validated the predictive ability of the PIS and IGPS model at 10-year survival (Figs. 4C for PIS and 4D for IPGS). The AUC value of PIS in GSE10846 dataset was 0.562 and the IGPS was 0.718, which confirmed that IGPS was a more robust and independent prognosticator than PIS.

## Discussion

Our study integrated the CIBERSORT algorithm and Lasso penalized Cox regression to reanalyze the gene expression dataset of 229 patients with DLBCL in TCGA. High numbers of TME-infiltrating resting NK cells combined with low numbers of activated NK cells were associated with a favorable prognosis in patients with DLBCL. A predictive prognostic risk model was developed to classify patients with DLBCL into two risk groups. We developed a prognostic immune-related gene-signature, contained upregulation of genes encoding CCL18, CCL4, CCL3, CCL23, CCL7, CCL13, CXCL10, and inflammatory cytokine IL-12A, molecules involved in chemokine signaling pathway, TLR signaling, and the cytokine–cytokine receptor interaction. These may contribute to the classification of the patients with DLBCL.

Cancer is a cellular disease caused by genetic mutations in a single cell type as well as an ecological disease involving the dynamic reprogramming of tumor cells and immune cells in the TME (Gajewski, Schreiber & Fu, 2013; Nicholas, Apollonio & Ramsay, 2016). The reprogramming of immune cells in the TME has provided crucial contributions to tumor progression and the suppression of anti-tumor immune responses. Despite IPI being an internationally accepted prognostic indicator, potential biomarkers for the systematic classification of DLBCL genomic heterogeneity have been improved by high-throughput biotechnology (Keane et al., 2017). Better comprehension of DLBCL heterogeneity could separate patients into more homogeneous subgroups for which potential candidates and therapeutic targets may be specifically developed in ways. CIBERSORT is a deconvolution method for complex tissues especially for human leukocyte subsets and is based on linear support vector regression (SVR) from gene expression profiles. We applied the Lasso regression model to obtain the predictive model for DLBCL patients to control the fitting of variables. According to the formula, the coefficient of the Tregs, M2 and M0 macrophages was significantly lower than that of the activated and resting NK cells, which explained that activated and resting NK cells dominated the role, also this was consistent with the survival results shown in Fig. 1C. Tregs and M2 and M0 macrophages contributed to the predictive ability of the model, which is attributed to the high coefficient and prognosis prediction superiority of the independent predictions by the two types of NK cells, as did activated and resting NK cells with independent predictive prognosis.

The phenotype of NK cells and macrophages is influenced by malignant B cells and contribute to the clinical sensitivity of programmed cell death ligand 1 (PD-L1) (Vari et al., 2018). NK cell dysfunction is prevalent in hematological cancer and is one of the mechanisms of tumor immune escape (Vo et al., 2018). A recent study using CIBERSORT with the GEO database found that a higher number of active NK cells indicated a poorer outcome (Ciavarella et al., 2018). Our findings that neither CD4+ T cells nor dendritic cell associated with prognosis of DLBCL may be due to the dynamic interactions among immune cells. In fact, the resting and activated phenotype of NK cells were represented as functional regulators with a more effective killing ability than the others (Cerwenka & Lanier, 2018). Hence, our observation of NK cells may provide a checkpoint modulator. Patients with DLBCL with a higher percentage of NK cells in the blood were associated with a greater cytotoxic ability and interferon production capability (Cox et al., 2015). Other immune cells also demonstrated a similar prognostic relationship in patients with DLBCL (Nicholas, Apollonio & Ramsay, 2016). These findings suggest that the reprogramming of NK cells and macrophages in the TME play an important prognostic role in tumor growth and metastasis.

GSEA was performed to better understand the two immunoscore types of DLBCL genetic characteristics and underlying molecular mechanisms and to identify the key genes and their biological functions. It is widely recognized that chemokines, particularly the abnormal expression of chemotactic cytokines in TME, are a major part of the crosstalk between tumor cells and TME (Shain, Dalton & Tao, 2015). Several chemokines have acted as potential markers or therapeutic targets for DLBCL spatiotemporal-regulated TME immune responses and influence cancer progression (Nagarsheth, Wicha & Zou, 2017). Consistent with our results, these associations have been observed between the prognoses and expressions of chemokines including CCL3, CCL4, CCL18, and CXCL10 in patients with DLBCL. The elevated levels of CCL3 and CCL4 are a signature hallmark in activated B-cell-like (ABC) DLBCL, as a category of DLBCL, and are a clear predictor of poor prognosis (Takahashi et al., 2015). The underlying molecular mechanism of high levels of CCL3 and CCL4 is the response to the B-cell receptor and NF-κB pathway activation (Kim et al., 2017; Takahashi et al., 2015). CCL18 showed similar results in DLBCL (Zhou et al., 2018). CXCL10 expression levels detected from the blood of patients with DLBCL were reported in three prospective studies, each including 185 (Ansell et al., 2012), 276 (Witzig et al., 2014) and 313 (Hong et al., 2017) patients, to correlate with higher IPI scores and significant inferior prognoses. However, CCL7, CCL13, and CCL23 were first identified in our study, suggesting the combination of these chemokines may be a new approach to assign PIS for patients with DLBCL. We pooled these immune-related genes into a single summary score based on a weighted formula to increase the robustness of the results and account for the possible misclassification of patients due to aberrant expression of one or a few genes. Similar to our validation dataset, a high level of resting NK cells and IGPS correlated with poor clinical outcomes. We used the voom method to convert the RNA-seq data into a form that could be comparable and suitable for the microarray-based method. Novel methods of normalization are still needed to deal with the integer counts of RNA-seq and continuous numeric of mircoarray due to the intrinsic heterogeneity caused by these two kinds of methods (Law et al., 2014). Therefore, we can conclude that resting NK cells and IGPS may play pivotal roles in the immune system for the prognosis of DLBCL patients.

Some limitations of this study should be acknowledged. Our findings were based on publicly available datasets containing incomplete clinical information and regions of tumor cores that may preclude accuracy. We followed several studies using similar methods to perform permutations with a random list to define every cell type in the LM22 signature (Zhang et al., 2019). Further studies of higher random permutations for the assessment of prognostic or predictive relationships from extensive datasets of DLBCL are still required. We were also missing the information on pre-treatment and chemoimmunotherapy in TCGA DLBCL corhort. Although our results do not provide therapeutic targets in DLBCL TME, we emphasized the need to enhance the prognostic functionality of predictive biomarkers or models to streamline clinical treatments. We believe that further large-scale, prospective cohort studies may determine the functional processes linked to definite infiltrating cell types within the TME and molecular variants to predict DLBCL prognoses.

## Conclusions

In summary, our findings show that different abundances of tumor-infiltrating immune cells can affect the clinical outcomes of DLBCL patients and molecular characteristics. We discovered that a prognostic immune-related gene signature may be superior than the prognostic benefits based on the PIS model with NK cells, Tregs, and macrophages. Advanced technologies should be used to improve prediction algorithms and the personalized management of DLBCL patients.

##  Supplemental Information

10.7717/peerj.9658/supp-1 Supplemental Information 1 Summary of the workflow in this study

10.7717/peerj.9658/supp-2 Supplemental Information 2 The percent of 22 tumor-infiltrating immune cells factions in DLBCL

10.7717/peerj.9658/supp-3 Supplemental Information 3 Dataset in the present study

10.7717/peerj.9658/supp-4 Supplemental Information 4 R codes in the present study

## Additional Information and Declarations

### Data Availability

The following information was supplied regarding data availability: The data that support the findings of this study are publicly available at The Cancer Genome Atlas (search terms: CTSP-DLBCL1, NCICCR-DLBCL) and the NCBI Gene Expression Omnibus (GSE10846). The R codes are available in the Supplemental Files.
