# Supplementary figures and images for: A prognostic gene model of immune cell infiltration in diffuse large B-cell lymphoma
Source: PeerJ. 2020 Aug 5;8:e9658. doi: 10.7717/peerj.9658 (PMC7414766; doi:10.7717/peerj.9658)

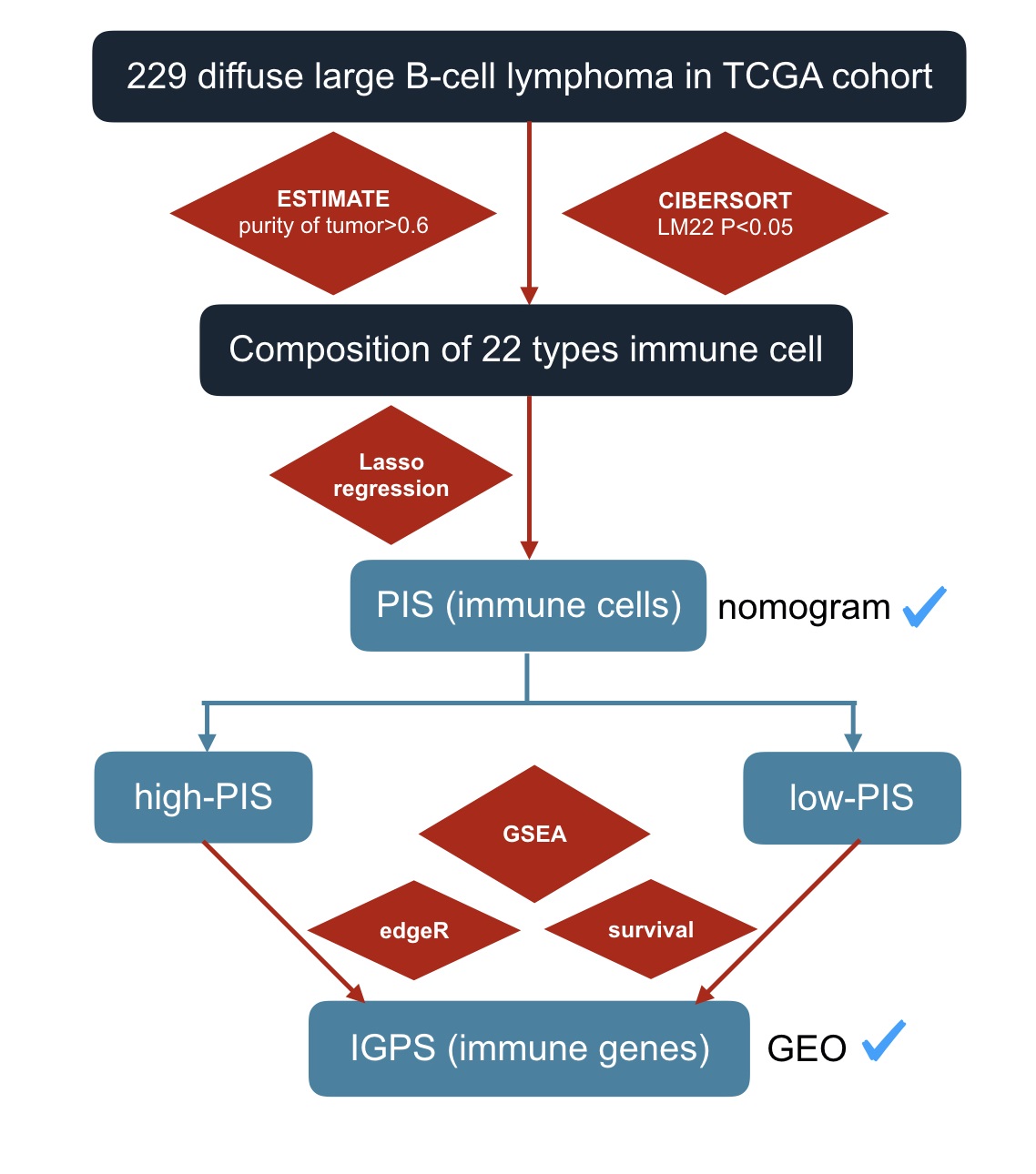

Supplement: Supplemental Information 1 [file peerj-08-9658-s001.jpg]

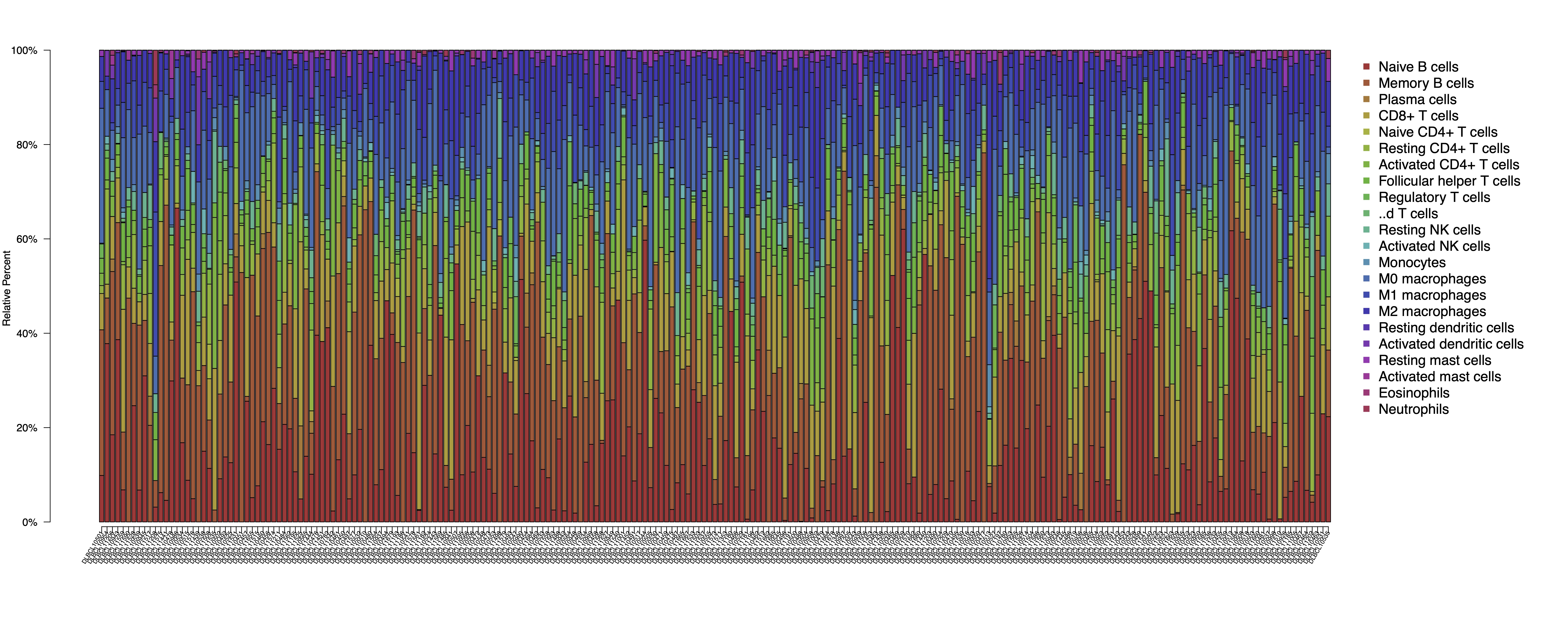

Supplement: Supplemental Information 2 [file peerj-08-9658-s002.jpg]
